# Microbial metabolite–mediated modulation of enteroendocrine and immune pathways by *Lactiplantibacillus pentosus* CECT 8039 in early diet-induced obesity

**DOI:** 10.3389/fendo.2026.1882392

**Published:** 2026-09-03

**Authors:** Carmen Veciana-Galindo, Eva Núñez-Delegido, Jorge González-Santos, Juan Agüera-Santos, Vicente Navarro-López, Ignacio Alberola-Jordá

**Affiliations:** 1 Biopartner SL, Alcoy, Alicante, Spain; 2 Research and Development Department, Bioithas SL, Alicante, Alicante, Spain; 3 Human Microbiota Research Group, Universidad Católica de Murcia (UCAM), Guadalupe, Murcia, Spain; 4 Health Sciences PhD Program, Universidad Católica de Murcia (UCAM), Guadalupe, Murcia, Spain

**Keywords:** adipokines, colonic fermentation, enteroendocrine signaling, glucagon-like peptide-1, high-fat diet, immunomodulation, probiotic, short-chain fatty acids

## Abstract

**Introduction:**

The gut microbiota–enteroendocrine axis contributes to the regulation of host energy homeostasis through microbial metabolites and satiety-related signaling. This study evaluated whether *Lactiplantibacillus pentosus* CECT 8039 (*L. pentosus* BB) modulates microbial fermentation, enteroendocrine responses, and metabolic and immune parameters associated with early diet-induced obesity.

**Methods:**

A dynamic multi-compartment colonic fermentation model was used to assess changes in cultivable microbial groups and short-chain fatty acid (SCFA) concentrations following probiotic treatment. Fermentation samples were subsequently evaluated in STC-1 enteroendocrine cells and a Caco-2/THP-1 co-culture model. In parallel, the metabolic and immunomodulatory effects of *L. pentosus* BB were assessed in mice fed a high-fat diet (HFD) for 28 days.

**Results:**

Probiotic treatment selectively modulated cultivable microbial groups, increasing lactic acid bacteria and decreasing *Clostridium* spp. across the three simulated colonic compartments, while other groups showed region-dependent changes. Acetate, propionate, and butyrate concentrations increased in all three compartments; in the ascending colon, they increased 2.53-, 7.34-, and 5.08-fold, respectively, relative to the end of stabilization. Fermentation samples collected after probiotic treatment increased GLP-1 release, reaching statistical significance in the transverse-colon condition, while *Pyy* mRNA expression increased significantly in the ascending and descending colon conditions and *Cck* expression increased significantly in all three compartments. *In vivo*, HFD-fed mice showed a 34.9 ± 5.5% body-weight gain, whereas probiotic supplementation was associated with a 13.2 ± 4.7% lower relative body-weight gain compared with the HFD control group. In HFD-fed mice, probiotic supplementation reduced circulating resistin by 25.8 ± 9.4% and leptin by 85.5 ± 20.1%, whereas the change in adiponectin was not statistically significant. Immune responses were heterogeneous and depended on the experimental context.

**Discussion:**

*L. pentosus* BB was associated with increased colonic SCFA concentrations, enhanced enteroendocrine responses, and partial attenuation of HFD-associated metabolic alterations. These findings support a functional association between probiotic-modulated microbial fermentation and host endocrine and metabolic responses, although direct SCFA-mediated causality remains to be established.

## 1 Introduction

Energy intake is tightly regulated by integrated neurohormonal networks that coordinate peripheral nutrient sensing with central appetite control (1). Enteroendocrine cells in the gastrointestinal tract secrete hormones such as glucagon-like peptide-1 (GLP-1), peptide YY (PYY) and cholecystokinin (CCK) in response to luminal nutrients. These hormones modulate gastric emptying, insulin secretion, and central appetite circuits, promoting satiety and limiting food intake (2, 3). Impaired secretion or signaling of these hormones is frequently observed in obesity and contributes to disrupted appetite regulation and positive energy balance over time (3).

Obesity is a complex metabolic disorder defined by excessive adiposity and associated with elevated risk for insulin resistance, dyslipidemia, type II diabetes mellitus and chronic low-grade inflammation (4). Beyond excess caloric intake, obesity involves perturbations in metabolic signaling pathways, including altered enteroendocrine function, disrupted adipokine profiles and sustained activation of inflammatory cascades (4, 5). These pathophysiological features underlie progression from early weight gain to established metabolic syndrome.

Recent evidence indicates that the gut microbiota—the dense and diverse microbial community inhabiting the gastrointestinal lumen—acts as a critical modulator of host energy metabolism and endocrine signaling. Host–diet–microbiome interactions have been shown to influence energy balance and metabolic regulation, highlighting the role of microbial ecosystems in metabolic health (6). Alterations in microbiota composition and metabolic activity (dysbiosis) have been associated with obesity and its metabolic complications, influencing nutrient harvest, intestinal barrier integrity and immune regulation (4, 7). Microbiota-derived metabolites, particularly short-chain fatty acids (SCFAs) such as acetate, propionate and butyrate, have emerged as key mediators of microbiota–host cross-talk (1, 8, 9). SCFAs interact with host G-protein-coupled receptors including FFAR2 and FFAR3 expressed on enteroendocrine and immune cells, stimulating secretion of anorexigenic hormones and modulating glucose and lipid metabolism (8, 10).

Probiotics, defined as live microorganisms that confer health benefits when administered in adequate amounts, are increasingly investigated for their capacity to modulate gut microbial ecosystems and enhance production of bioactive metabolites such as SCFAs (11). Supplementation with probiotics may therefore influence host satiety pathways and metabolic homeostasis via microbiota-mediated mechanisms, representing a promising strategy to prevent or attenuate early metabolic disturbances in the context of nutrient excess.

Given the emerging role of microbial metabolites in the regulation of host energy homeostasis, the present study aimed to evaluate whether supplementation with *Lactiplantibacillus pentosus* CECT 8039 (*L. pentosus* BB) modulates enteroendocrine signaling during early diet-induced obesity. The study investigated the strain’s capacity to enhance microbial SCFA production and influence satiety-related hormonal pathways. We hypothesized that probiotic-driven increases in endogenous SCFA production stimulate anorexigenic hormone expression and improve metabolic and inflammatory parameters associated with early obesity, thereby supporting a microbiota–metabolite–host axis involved in appetite regulation and energy balance.

## 2 Materials and methods

### 2.1 Bacterial strain and preparation

The probiotic strain used in this study, *L. pentosus* BB (CECT 8039), was supplied and used in freeze-dried form in both the *in vitro* and *in vivo* experiments. Prior to use, batch-specific viability was determined by plate counting on de Man–Rogosa–Sharpe (MRS) agar under anaerobic conditions at 37 °C for 48 h. Viable cell counts were expressed as colony-forming units (CFU) per gram. The viable count and administered dose used in each experimental model are described in the corresponding sections.

### 2.2 *In vitro* colonic fermentation system

The modulatory effect of *L. pentosus* BB on gut microbiota composition and metabolic activity was assessed using the ColonSim^®^ platform (AINIA, Valencia, Spain), a dynamic, multi-compartment gastrointestinal simulation system reproducing the physicochemical and transit conditions of the upper gastrointestinal tract and the three main colonic regions.

The system comprised five sequentially connected, temperature-controlled bioreactors simulating the stomach (R1; 260 mL; mean retention time 2 h), small intestine (R2; 410 mL; 6 h), ascending colon (R3; 1000 mL; 20 h), transverse colon (R4; 1600 mL; 32 h), and descending colon (R5; 1200 mL; 24 h). The overall gastrointestinal transit time was approximately 3.5 days. The stomach and small-intestinal compartments operated in a semicontinuous mode, whereas the colonic reactors operated under continuous-flow conditions.

The temperature was maintained at 37 °C throughout the gastrointestinal simulation. The probiotic preparation was initially mixed with artificial saliva containing electrolytes and α-amylase (9,600 U) and subsequently transferred to the gastric compartment. Gastric pH gradually decreased from 4.5 to approximately 2.0 during the 120-min gastric phase, while gastric electrolyte solution containing pepsin and lipase was supplied. Gastrointestinal transit was simulated using computer-controlled valves. In the small-intestinal compartment, pH was maintained at 6.5–7.0, and intestinal electrolyte solution, pancreatin, and bile extract were added.

In the colonic reactors, pH was continuously monitored and maintained within region-specific physiological ranges: 5.5–6.0 in the ascending colon, 6.0–6.4 in the transverse colon, and 6.4–6.8 in the descending colon. Anaerobic conditions were maintained by flushing all reactors with N_2_ for 15 min daily.

The colonic culture medium was based on that described by Molly et al. (12, 13 and contained carbohydrates, including pectin, xylan, starch, and glucose, together with yeast extract, peptone, mucin, cysteine, Tween 80, mineral salts, trace elements, and vitamins.

### 2.3 Fecal inoculum and stabilization

Fresh fecal samples were obtained from two healthy adult donors who were non-smokers, had no history of intestinal disease, and had not received antibiotic treatment or probiotic supplementation during the three months preceding the study. Samples from the two donors were used to introduce biological variability into the inoculum and obtain a microbial community more representative of the adult human intestinal microbiota. Fecal sample collection was performed following informed consent from donors and in accordance with institutional ethical guidelines.

Samples were collected using specific anaerobic systems (BD GasPak^®^) and processed on the same day of collection. A 20% (w/v) fecal suspension was prepared and used to inoculate the ascending, transverse, and descending colon reactors.

Following inoculation, the system underwent a 9-day stabilization period to allow microbial communities to adapt to the *in vitro* conditions and reach stable levels within each colonic region.

### 2.4 *In vitro* treatment phase

Following the 9-day stabilization period, a 14-day treatment phase was initiated. The freeze-dried *L. pentosus* BB preparation was administered once daily at a dose of 10 g in 200 mL of fresh culture medium. The viable count of the freeze-dried preparation was 5.40 × 10^7^ CFU/g, corresponding to approximately 5.40 × 10^8^ CFU per daily administration, within the intended daily range of 10^8^–10^9^ CFU. Two additional 200-mL additions of fresh culture medium without probiotic were supplied daily to the gastric reactor. During the basal period, 200 mL of fresh culture medium were supplied to the gastric reactor three times daily.

Microbial composition was assessed using samples aseptically collected from the ascending, transverse, and descending colon reactors. Samples (10 mL) were collected on experimental days 5 and 9, during the stabilization period, and on days 14 and 22, during the treatment period. Viable counts of five representative microbial groups were determined using culture-based methods: *Bifidobacterium* spp. on TOS-propionate agar, lactic acid bacteria on MRS agar, Enterobacteriaceae on VRBD agar, *Clostridium* spp. on TSC agar, and total anaerobes on Schaedler agar. Plates were incubated at 37 °C for 24–72 h under the atmosphere appropriate for each microbial group, and results were expressed as log CFU/mL.

Microbial metabolic activity was assessed by quantifying acetic, propionic, and butyric acids. Samples (50 mL) were collected from each colonic reactor at the end of the stabilization and treatment periods, divided into two aliquots, and stored at −20 °C until analysis. SCFAs were extracted by liquid–liquid extraction with diethyl ether, followed by filtration of the resulting extract. Quantification was performed by gas chromatography coupled to flame-ionization detection (GC-FID) using an HP-FFAP column. SCFA concentrations were determined from calibration curves using capric acid as the internal standard and were expressed as mg/kg of colonic medium.

Fermentation samples intended for functional cellular assays were centrifuged and filtered before application to the cellular models. Sample dilution was performed using the assay-specific medium or buffer described below.

### 2.5 Enteroendocrine cell assays

The potential enteroendocrine activity of colonic fermentation samples was evaluated using the murine enteroendocrine STC-1 cell line (ATCC^®^ CRL-3254™). Cells were maintained in Dulbecco’s modified Eagle medium (DMEM) supplemented with 10% fetal bovine serum (FBS), penicillin (100 U/mL), and streptomycin (100 µg/mL), at 37 °C in a humidified atmosphere containing 5% CO_2_.

The appropriate sample dilution was selected by preliminary cytotoxicity testing in STC-1 cells. Serial dilutions of the fermentation samples were evaluated using 80% cell viability as the acceptance threshold. Based on these results, samples from the *L. pentosus* BB fermentation experiment were applied at a 1:16 dilution. Fermentation samples were diluted in HEPES buffer containing 140 mM NaCl, 4.5 mM KCl, 1.2 mM CaCl_2_, 1.2 mM MgCl_2_, and 20 mM HEPES.

For the functional assays, STC-1 cells were seeded in 96-well plates and cultured at 37 °C and 5% CO_2_ until reaching 70–80% confluence. Cells were exposed to the diluted fermentation samples for 3 h. After treatment, culture supernatants were collected, centrifuged, and stored frozen until hormone analysis, whereas the cell fraction was recovered for gene-expression analysis.

Glucagon-like peptide-1 (GLP-1) release into the culture supernatant was quantified by enzyme-linked immunosorbent assay (ELISA). Peptide YY (PYY) was also initially evaluated by ELISA; however, the measured concentrations were below the assay detection limit. Consequently, PYY was evaluated at the transcriptional level, together with cholecystokinin (CCK), by quantitative real-time PCR.

For the GLP-1 assay, results were expressed relative to the sample diluent, HEPES buffer. DMEM was used as the basal medium control, whereas DMEM supplemented with FBS was included as a positive control for GLP-1 release. The assay comprised two independent experiments with three replicates per condition.

For gene-expression analyses, total RNA was extracted using the RNeasy Mini Kit (Qiagen, Germany) according to the manufacturer’s protocol, and cDNA was synthesized from 1 µg of total RNA using the PrimeScript™ RT reagent kit (Takara Bio, Japan). Relative mRNA expression of *Pyy* and *Cck* was determined by RT-qPCR using a 7300 Real-Time PCR System (Applied Biosystems) and commercially available TaqMan Gene Expression Assays (Thermo Fisher Scientific, Waltham, MA, USA): *Pyy* (Mm00520716_g1), *Cck* (Mm00446170_m1), and *Actb* (Mm01205647_g1). Amplification was performed using universal cycling conditions consisting of 50 °C for 2 min and 95 °C for 10 min, followed by 40 cycles of 95 °C for 15 s and 60 °C for 1 min. *Actb* was used as the endogenous reference gene, and relative gene expression was calculated using the 2^−ΔΔ^Ct method. Results were expressed as fold change relative to the corresponding reference condition. The assays comprised two independent experiments with three replicates per experiment.

### 2.6 Intestinal epithelial–macrophage co-culture assay

The immunomodulatory activity of colonic fermentation samples was evaluated using a Caco-2/THP-1 co-culture model. Caco-2 cells were cultured on Transwell inserts for 21 days to obtain a differentiated intestinal epithelial monolayer, while THP-1 monocytes were differentiated into macrophage-like cells using phorbol 12-myristate 13-acetate (PMA). The Caco-2 inserts were then placed above the differentiated THP-1 cells to establish the co-culture system.

Samples collected from the ascending, transverse, and descending colon compartments at the end of the stabilization and probiotic-treatment phases were centrifuged, filtered, and diluted 1:4 in RPMI-1640 medium (ATCC 30-2001) supplemented with 10% FBS, 1% penicillin–streptomycin, and 0.05 mM 2-mercaptoethanol. This dilution had previously been selected by cytotoxicity testing to maintain cell viability above 80%. Samples were applied to the Caco-2 compartment, and co-cultures were maintained for 24 h under basal conditions or stimulated with lipopolysaccharide (LPS; 10 ng/mL; Sigma-Aldrich, St. Louis, MO, USA), added 2 h after sample application.

At the end of the exposure period, THP-1 cells were collected for gene-expression analysis. Relative mRNA expression of *IL6* and *PTGS2* (COX-2) was determined by RT-qPCR using commercially available TaqMan Gene Expression Assays (Thermo Fisher Scientific, Waltham, MA, USA): *IL6* (Hs00174131_m1), *PTGS2* (Hs00153133_m1), and *ACTB* (4326315E). Amplification conditions and relative expression calculations were performed as previously described. The assays comprised two independent experiments with three replicates per condition.

### 2.7 Animals and housing conditions

Male C57BL/6J mice (8 weeks of age) were obtained from Charles River Laboratories (France). Upon arrival, animals were acclimatized for two weeks prior to the initiation of the experimental protocol. Throughout the study, mice were maintained under controlled environmental conditions (22 ± 2 °C; 50–60% relative humidity; 12 h light/12 h dark cycle) and housed in individually ventilated cages with *ad libitum* access to food and water.

All experimental procedures were conducted in accordance with European Directive 2010/63/EU on the protection of animals used for scientific purposes and were approved by the corresponding Institutional Animal Care and Use Committee [Animal Testing Ethics Committee (CEEA) at La Fe Health Research Institute (IIS La Fe) (Valencia, Spain); reference: CEEA.IP.JLMS.#1-2-2016].

At the end of the 28-day intervention period, animals were euthanized by authorized personnel. Following a 16-hour fasting period, mice were individually placed in an induction chamber and anesthetized with inhaled isoflurane until a deep surgical plane of anesthesia was reached. The depth of anesthesia was assessed by the absence of response to external stimuli. Once deep anesthesia was confirmed, mice were euthanized by cervical dislocation. Death was verified by confirming the absence of vital signs.

Immediately thereafter, a systematic necropsy was performed. The liver and spleen were aseptically excised, rinsed in ice-cold sterile PBS to remove residual blood, gently blotted dry, and weighed using a calibrated analytical balance. Absolute organ weights were recorded, and relative organ weights were calculated as organ-to-body weight ratios to normalize for inter-animal variability.

Macroscopic examination was performed to identify potential gross morphological alterations with particular attention to indicators of hepatotoxicity and splenomegaly. These features were considered relevant markers of systemic toxicity or inflammatory responses associated with dietary and probiotic interventions.

### 2.8 Dietary intervention and experimental groups

Following acclimatization, animals were randomly assigned to experimental groups (n = 6 per group per experimental set). Mice received either a standard chow diet or a high-fat diet (HFD; SAFE^®^, Ref. 260 HF) for 4 weeks. The standard chow diet provided approximately 10% of total energy from fat, 20% from protein and 70% from carbohydrates (energy density ~3.1 kcal/g). The HFD was formulated to induce a pre-obese phenotype and provided 60% of total energy from fat, 20% from protein and 20% from carbohydrates (energy density ~5.2 kcal/g).

The probiotic strain (*L. pentosus* BB) was administered once daily by oral gavage at a dose of 1 × 10^9^ CFU per mouse in a fixed volume of vehicle throughout the 28-day intervention period. Control groups received the corresponding vehicle under identical conditions.

The experimental design included four groups: (i) standard chow diet (Control), (ii) HFD, (iii) Probiotic (standard chow diet supplemented with the probiotic), and (iv) Probiotic + HFD (high-fat diet supplemented with the probiotic). This design allowed evaluation of the independent and combined effects of diet and probiotic supplementation on metabolic and immunological outcomes in a pre-obese murine model.

### 2.9 Monitoring of physiological parameters

Body weight was recorded at baseline (day 0) and subsequently on a weekly basis (days 7, 14, 21, and 28) using a calibrated analytical balance. Body weight gain was calculated as both the absolute and relative change from baseline values.

Food intake was monitored throughout the intervention period to detect potential differences in caloric consumption among experimental groups. Feed consumption was quantified by measuring the amount of diet provided and the residual food at defined time points, allowing estimation of mean daily intake at the cage level.

Animal health and welfare were assessed daily through systematic observation of clinical signs, grooming behavior, posture, and spontaneous locomotor activity. Any signs of distress, abnormal behavior, or adverse effects were recorded and managed in accordance with institutional animal care guidelines.

### 2.10 Hormonal and biochemical measurements

Blood samples were collected in heparinized tubes (Heparin; Sigma-Aldrich, St. Louis, MO, USA) at baseline (day 0), mid-intervention (day 14), and at the end of the experimental period (day 28). For plasma separation, blood samples were centrifuged at 1,000 × g for 10 min at 4 °C. The resulting plasma was carefully aliquoted to minimize hemolysis and stored at −80 °C until further biochemical and hormonal analyses.

Plasma concentrations of leptin, resistin and adiponectin were quantified using commercially available enzyme-linked immunosorbent assay (ELISA) kits (SPI Bio, France; R&D Systems, USA), according to manufacturers’ instructions. All samples and standards were assayed in duplicate to ensure analytical reliability.

Absorbance was measured at the recommended wavelength using a calibrated microplate reader. Analyte concentrations were calculated from standard curves generated for each assay using four-parameter logistic regression. Results were expressed in ng/mL according to the specifications provided by each kit, and subsequently normalized to the corresponding control group.

### 2.11 Evaluation of immunomodulatory activity

At study termination, spleens were aseptically excised, weighed to assess potential splenomegaly, and processed immediately for downstream immunological analyses. Single-cell suspensions were generated by mechanical dissociation through sterile 70 µm cell strainers in cold PBS (Invitrogen, Renfrewshire, United Kingdom). Erythrocytes were removed using an ammonium chloride-based red blood cell lysis buffer (Sigma-Aldrich, St. Louis, MO, USA), followed by washing and resuspension in complete culture medium. Cell viability was assessed by Trypan Blue exclusion (Bio-Rad, Hercules, CA, USA).

Splenocytes were cultured in RPMI-1640 medium (Invitrogen, Renfrewshire, United Kingdom) supplemented with 10% heat-inactivated fetal bovine serum (Sigma-Aldrich, St. Louis, MO, USA), 1% penicillin–streptomycin (Invitrogen, Renfrewshire, United Kingdom), and L-glutamine (Invitrogen, Renfrewshire, United Kingdom), and maintained at 37 °C in a humidified incubator with 5% CO_2_. To evaluate inflammatory responsiveness, cells were stimulated with LPS for 24 h, while unstimulated cultures served as basal controls.

RNA extraction and cDNA synthesis were performed as previously described. Quantitative PCR was performed using a standard SYBR Green-based protocol. The following primer pairs were used (forward/reverse, 5′–3′): *Tnf* (5′-CCACCACGCTCTTCTGTCTA-3′/5′-CAGGCTTGTCACTCGAATTTTGA-3′), *Nfkb1* (5′-CCCACAGGTCAAAATTTGCAACT-3′/5′-TTGGGTCCTGCTGTTACGGT-3′), and *Actb*, used as the reference gene, (5′-GGCTGTATTCCCCTCCATCG-3′/5′-CCAGTTGGTAACAATGCCATGT-3′). All primers were purchased from Sigma-Aldrich. Relative gene expression was calculated using the 2^−ΔΔCt^ method and expressed as fold change relative to the corresponding control group.

### 2.12 Statistical analysis

Data from the dynamic colonic fermentation experiment were analyzed descriptively because the three colonic reactors represented distinct anatomical compartments within a single experimental system and were not independent biological replicates. Accordingly, no inferential statistical analyses were performed for microbial counts or SCFA concentrations.

Data from cellular and animal experiments are presented as mean ± SD or mean ± SEM, as specified in the corresponding figure legends. Pairwise comparisons between experimental groups were performed using two-tailed unpaired Student’s *t*-tests. Statistical significance was set at *p* < 0.05. Analyses were conducted using GraphPad Prism version 7 (GraphPad Software, San Diego, CA, USA).

## 3 Results

### 3.1 Probiotic treatment selectively modulates cultivable microbial groups

To assess the effect of *L. pentosus* BB on the cultivable microbial community, viable counts of five representative bacterial groups were determined in the ascending, transverse, and descending colon compartments (**Table 1**).

**Table 1:** Viable counts of selected cultivable microbial groups at the end of the stabilization and probiotic-treatment phases in the dynamic colonic fermentation system.

Colonic compartment	Microbial group	Stabilization (log CFU/mL)	Treatment (log CFU/mL)	Change (log CFU/mL)
ASCENDING COLON	*Bifidobacterium* spp.	5.52	4.99	−0.53
	Lactic acid bacteria	3.90	6.73	+2.83
	Enterobacteriaceae	5.52	6.56	+1.04
	*Clostridium* spp.	6.27	2.97	−3.30
	Total anaerobes	6.27	7.70	+1.43
TRANSVERSE COLON	*Bifidobacterium* spp.	5.39	4.89	−0.50
	Lactic acid bacteria	5.00	6.17	+1.17
	Enterobacteriaceae	6.29	6.48	+0.19
	*Clostridium* spp.	6.40	4.60	−1.80
	Total anaerobes	7.20	8.12	+0.92
DESCENDING COLON	*Bifidobacterium* spp.	6.15	5.46	−0.69
	Lactic acid bacteria	5.43	6.23	+0.80
	Enterobacteriaceae	6.88	5.60	−1.28
	*Clostridium* spp.	6.00	5.52	−0.48
	Total anaerobes	7.52	7.66	+0.14

Values are expressed as log₁₀ CFU/mL and correspond to selective culture-based enumeration in samples collected from the ascending, transverse, and descending colon compartments. Change was calculated as the viable count at the end of treatment minus the count at the end of stabilization within the corresponding colonic compartment. The three colonic reactors represented anatomically distinct compartments within a single dynamic fermentation system and were not considered independent biological replicates. Therefore, the results are presented descriptively, and no inferential statistical analysis was performed.

At the end of the treatment period, lactic acid bacteria increased in all three colonic compartments, whereas *Clostridium* spp. counts decreased. Total anaerobic counts remained stable or increased, indicating that these selective changes were not accompanied by an overall loss of cultivable anaerobes. *Bifidobacterium* spp. showed modest reductions of less than 1 log CFU/mL, while Enterobacteriaceae displayed compartment-specific changes, increasing in the ascending colon, remaining relatively stable in the transverse colon, and decreasing in the descending colon.

Overall, these findings indicate that *L. pentosus* BB selectively modulated representative cultivable microbial groups, although the magnitude and direction of some changes varied among colonic regions.

### 3.2 SCFA concentrations increase during the probiotic-treatment phase

To determine whether *L. pentosus* BB modulates microbial metabolic activity, short-chain fatty acid (SCFA) concentrations were measured in each compartment of the dynamic *in vitro* colonic fermentation system at the end of the stabilization phase and following the treatment phase.

SCFA concentrations were higher at the end of the probiotic-treatment phase than at the end of stabilization in all three colonic compartments. Acetate increased by 2.53-, 1.60-, and 1.39-fold in the ascending, transverse, and descending colon compartments, respectively. Propionate showed the largest relative increase, reaching 7.34-, 3.36-, and 3.32-fold, while butyrate increased by 5.08-, 1.67-, and 1.69-fold, respectively (**Table 2**).

**Table 2:** Short-chain fatty acid concentrations at the end of the stabilization and probiotic-treatment phases in the dynamic colonic fermentation system.

Colonic compartment	SCFA	Stabilization (mg/kg)	Treatment (mg/kg)	Fold change
Ascending colon (R3)	Acetate	1127	2847	2.53
	Propionate	266	1952	7.34
	Butyrate	80	406	5.08
Transverse colon (R4)	Acetate	2029	3252	1.60
	Propionate	596	2003	3.36
	Butyrate	729	1218	1.67
Descending colon (R5)	Acetate	2305	3208	1.39
	Propionate	589	1954	3.32
	Butyrate	744	1258	1.69

Acetate, propionate, and butyrate concentrations were determined in samples collected from the ascending (R3), transverse (R4), and descending (R5) colon compartments and are expressed as mg/kg. Fold change was calculated by dividing the concentration measured at the end of treatment by the corresponding concentration measured at the end of stabilization. The three colonic reactors represented anatomically distinct compartments within a single dynamic fermentation system and were not considered independent biological replicates. Therefore, the results are presented descriptively and no inferential statistical analysis was performed.

### 3.3 Increased SCFA production is associated with upregulation of enteroendocrine markers

To determine whether the metabolic changes observed during colonic fermentation were associated with modulation of enteroendocrine signaling, key satiety-related hormones were evaluated following treatment with fermentation-derived products. GLP-1 release was assessed by ELISA, while *Pyy* and *Cck* mRNA expression was assessed by RT-qPCR.

**Figure 1:**
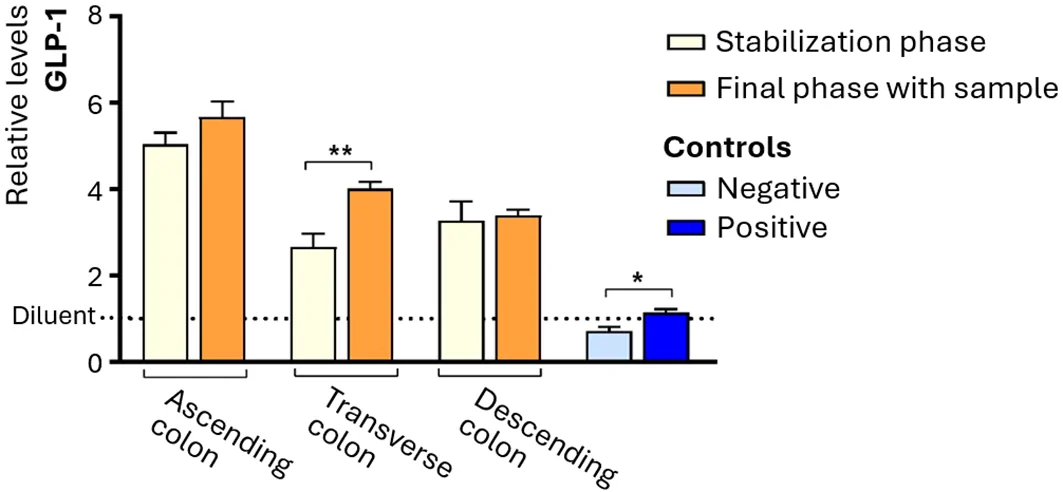
Glucagon-like peptide-1 (GLP-1) release by STC-1 cells following exposure to colonic fermentation samples. STC-1 cells were exposed for 3 h to samples collected from the ascending (R3), transverse (R4), and descending (R5) colon compartments at the end of the stabilization phase and at the end of the 14-day treatment phase with *Lactiplantibacillus pentosus* BB. Fermentation samples were applied at a 1:16 dilution. GLP-1 release into the culture medium was quantified by ELISA and expressed relative to the HEPES diluent control. DMEM was included as the basal medium control, whereas DMEM supplemented with fetal bovine serum (DMEM + FBS) was used as a positive control for GLP-1 release. Data are presented as mean ± SEM from two independent experiments, each comprising three replicate wells per condition. Asterisks indicate statistically significant differences between stabilization and treatment samples from the corresponding colonic compartment (*p ≤ 0.05; **p ≤ 0.01).

Treatment with samples collected at the end of the probiotic-treatment phase increased GLP-1 release across all colonic compartments; however, this increase reached statistical significance only for samples originating from the transverse colon (**Figure 1**).

**Figure 2:**
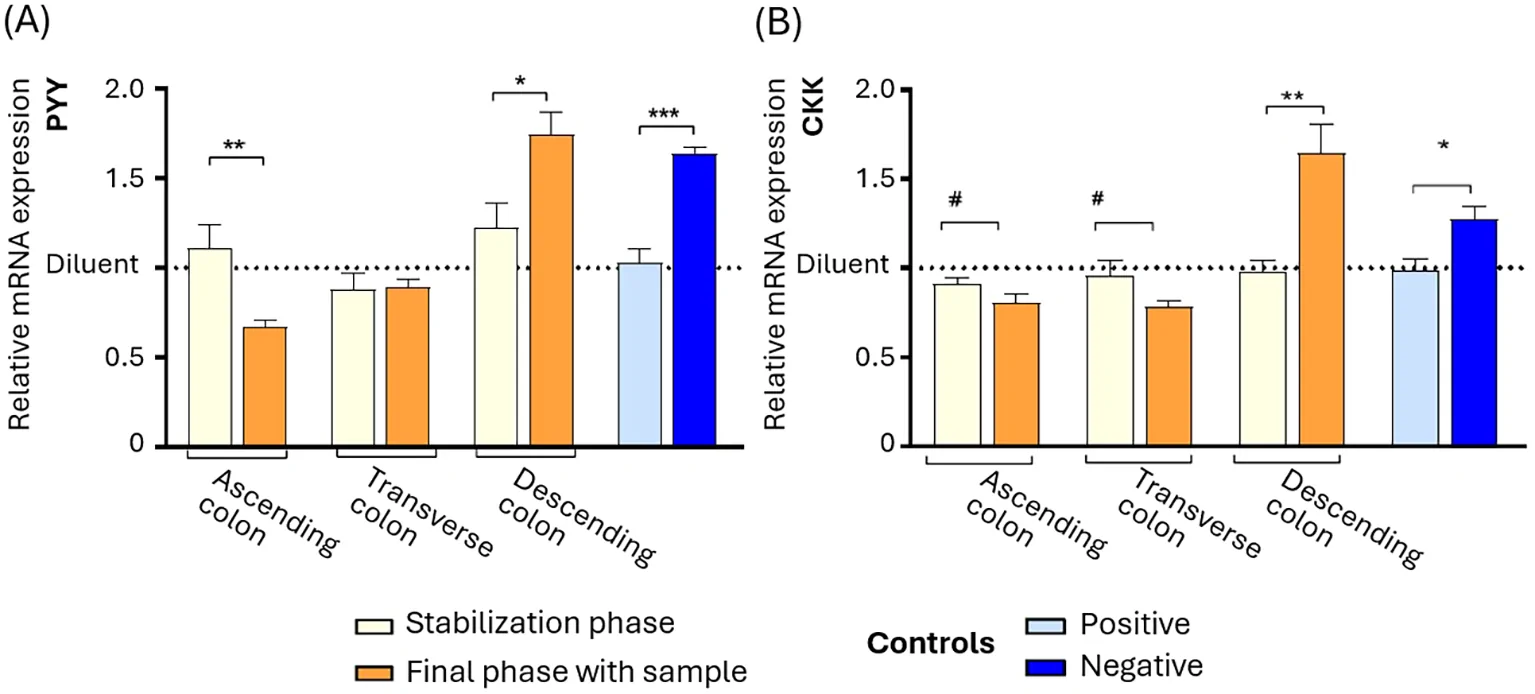
Relative expression of enteroendocrine markers in STC-1 cells following exposure to colonic fermentation samples. Relative mRNA expression of **(A)** peptide YY (*Pyy*) and **(B)** cholecystokinin (*Cck*) was determined in STC-1 cells exposed for 3 h to samples collected from the ascending (R3), transverse (R4), and descending (R5) colon compartments at the end of the stabilization phase and at the end of the 14-day treatment phase with *Lactiplantibacillus pentosus* BB. Fermentation samples were applied at a 1:16 dilution. Gene expression was determined by RT-qPCR, normalized to β-actin (*Actb*), and calculated using the 2^−ΔΔCt^ method. Results are expressed as fold change relative to the corresponding HEPES diluent control. Data are presented as mean ± SEM from two independent experiments, each comprising three replicate wells per condition. Asterisks indicate statistically significant differences between stabilization and treatment samples from the corresponding colonic compartment: * *p* ≤ 0.05, ** *p* ≤0.01, *** *p* ≤0.001.

A more consistent modulation was observed for *Pyy*. Exposure to fermentation supernatants resulted in increased relative mRNA expression in all compartments, with statistically significant upregulation detected for samples derived from both the ascending and descending colon reactors (**Figure 2A**). Similarly, *Cck* expression was higher after treatment in all three colonic compartments following treatment with probiotic-derived fermentation products. Increased relative mRNA expression was detected in samples originating from all colonic compartments, reaching statistical significance in the ascending, transverse, and descending colon conditions (**Figure 2B**).

### 3.4 Probiotic supplementation attenuates high-fat diet–induced weight gain

**Figure 3:**
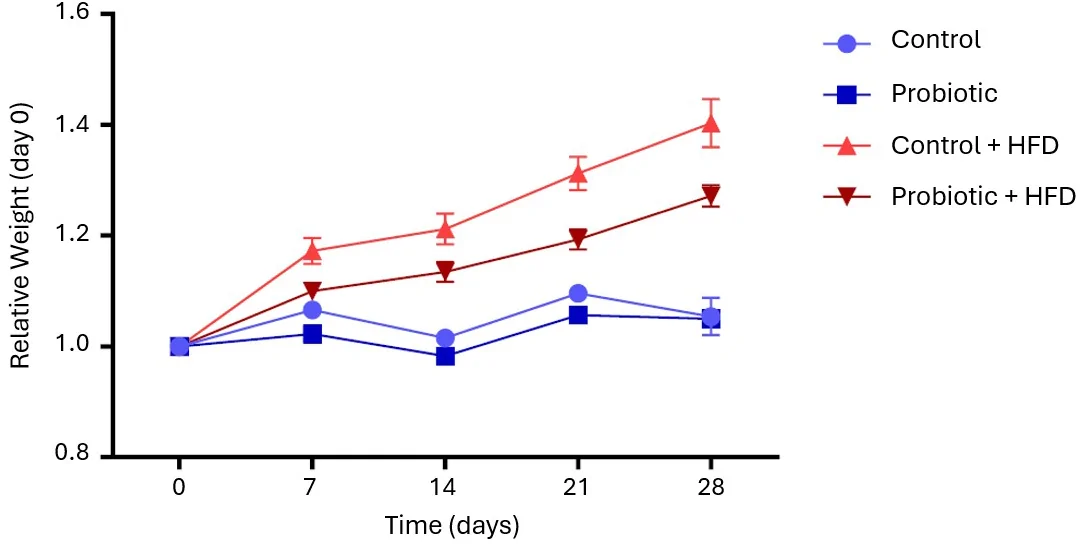
Body-weight evolution during the 28-day dietary intervention. Male C57BL/6J mice received a standard diet with vehicle (Control), a standard diet supplemented with *Lactiplantibacillus pentosus* BB (Probiotic), a high-fat diet with vehicle (Control + HFD), or a high-fat diet supplemented with the probiotic (Probiotic + HFD). Body weight was recorded at baseline and on days 7, 14, 21, and 28 and is expressed as the percentage change from each animal’s baseline body weight. Data are presented as mean ± SD. Each animal was considered an independent biological replicate; *n* = 6 animals per group.

Body weight was monitored weekly throughout the 28-day intervention to evaluate the effects of probiotic supplementation on weight gain during early diet-induced obesity (**Figure 3**). As expected, mice fed the high-fat diet (Control + HFD) exhibited a significant increase in body weight compared with animals receiving the standard diet (Control). After 28 days of dietary intervention, the Control+HFD group showed a weight gain of 34.9 ± 5.5% relative to baseline values, confirming the effectiveness of the diet-induced obesity model (*p* < 0.001).

Administration of *L. pentosus* BB in animals receiving the standard diet did not produce significant changes in body weight compared with the Control group, indicating that probiotic supplementation alone did not alter weight dynamics under normal dietary conditions.

In contrast, probiotic supplementation markedly attenuated weight gain in animals exposed to the HFD. Mice receiving both the probiotic and the HFD (Probiotic + HFD) showed a 13.2 ± 4.7% lower relative body-weight gain than the Control + HFD group at the end of the intervention period (*p* = 0.0107).

### 3.5 Modulation of adipokine profile under HFD conditions

**Figure 4:**
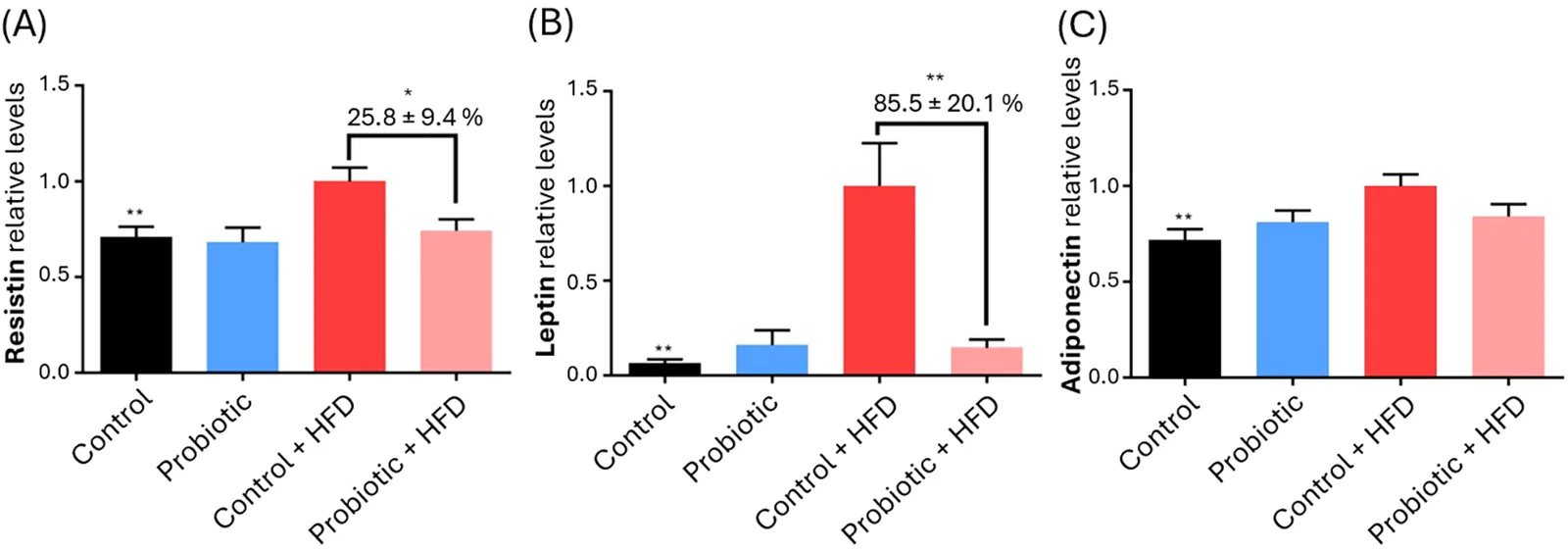
Circulating adipokine levels after the 28-day dietary intervention. Plasma levels of **(A)** resistin, **(B)** leptin, and **(C)** adiponectin were measured in mice receiving a standard diet with vehicle (Control), a standard diet supplemented with *Lactiplantibacillus pentosus* BB (Probiotic), a high-fat diet with vehicle (Control + HFD), or a high-fat diet supplemented with the probiotic (Probiotic + HFD). Results are expressed as relative values normalized to reference group (Control + HFD). Data are presented as mean ± SD. Each animal was considered an independent biological replicate; *n* = 6 per group. Pairwise comparisons were performed using two-tailed unpaired Student’s *t*-tests. Brackets indicate the groups included in each comparison: *p ≤ 0.05 and **p ≤ 0.01.

To further evaluate the metabolic impact of probiotic supplementation during early diet-induced obesity, circulating levels of key adipokines involved in energy homeostasis were quantified, including leptin, resistin, and adiponectin (**Figure 4**).

As expected, consumption of the high-fat diet (Control + HFD) significantly altered the adipokine profile compared with the control group. Plasma resistin levels increased by 41.5 ± 13.0%, while leptin concentrations exhibited a marked elevation, reaching 14.6 ± 3.2-fold higher levels than those observed in the Control group. These changes are consistent with the metabolic dysregulation typically associated with high-fat diet–induced obesity.

Probiotic supplementation in animals maintained on the standard diet did not produce significant differences in circulating adipokine levels relative to the Control group, indicating that administration of *L. pentosus* BB does not significantly alter adipokine homeostasis under normal dietary conditions.

In contrast, probiotic supplementation attenuated the adipokine alterations induced by the high-fat diet. In the Probiotic + HFD group, plasma resistin levels decreased by 25.8 ± 9.4% compared with the Control + HFD group. Similarly, leptin concentrations were significantly reduced by 85.5 ± 20.1%, indicating a strong attenuation of HFD-associated hyperleptinemia.

Regarding adiponectin, mice fed the high-fat diet showed a 39.4 ± 11.6% increase relative to the Control group. Probiotic supplementation in the HFD-fed animals resulted in a 15.9 ± 8.9% decrease compared with the Control + HFD group; however, this change did not reach statistical significance.

Together, these results indicate that supplementation with *L. pentosus* BB modulates the adipokine profile under high-fat diet conditions, particularly by reducing circulating resistin and leptin levels, suggesting a beneficial effect on metabolic signaling associated with early obesity.

### 3.6 Immunomodulatory effects *in vitro* and *ex vivo*

#### 3.6.1 Modulation of inflammatory markers in an intestinal epithelial–macrophage co-culture model

**Figure 5:**
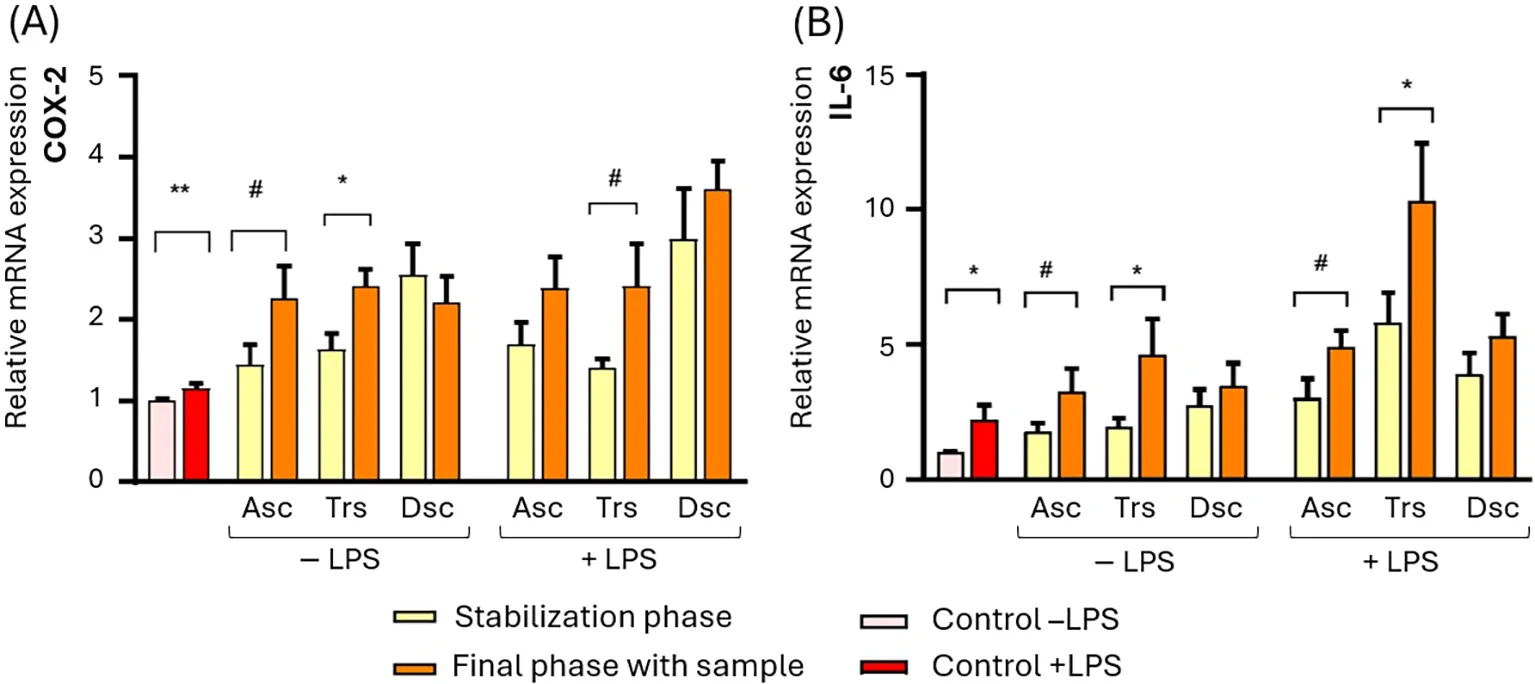
Expression of inflammatory markers in the Caco-2/THP-1 co-culture model following exposure to colonic fermentation samples. Relative mRNA expression of **(A)** cyclooxygenase-2 (*PTGS2*) and **(B)** interleukin-6 (*IL6*) was determined in THP-1 macrophage-like cells co-cultured with differentiated Caco-2 intestinal epithelial cells. Samples collected from the ascending (Asc; R3), transverse (Trs; R4), and descending (Dsc; R5) colon compartments at the end of stabilization and at the end of the probiotic-treatment phase were applied to the Caco-2 compartment at a 1:4 dilution. Co-cultures were maintained under basal conditions (−LPS) or stimulated with lipopolysaccharide (+LPS; 10 ng/mL), added 2 h after sample application, for a total exposure period of 24 h. Gene expression was determined by RT-qPCR, normalized to β-actin (*ACTB*), and calculated using the 2^−ΔΔCt^ method. Results are expressed as fold change relative to the corresponding sample diluent control. Data are presented as mean ± SD from two independent experiments, each comprising three replicate wells per condition. Symbols indicate comparisons between samples collected at the end of the probiotic-treatment phase and those collected at the end of stabilization within the same colonic compartment: ^#^*p* ≤ 0.1; *p ≤ 0.05; **p ≤ 0.01. # non-significant p-value.

To investigate whether metabolites generated during colonic fermentation exert immunomodulatory effects at the intestinal level, fermentation-derived digests were tested in a Caco-2/THP-1 co-culture model. Cells were exposed to fermentation supernatants and subsequently stimulated with LPS to induce an inflammatory response. The relative mRNA expression of *IL6* and *PTGS2* (COX-2) was evaluated (**Figure 5**).

In the absence of LPS stimulation, treatment with fermentation-derived samples resulted in a significant increase in *PTGS2* mRNA expression (**Figure 5A**) in cells exposed to digests originating from the ascending colon. A similar trend toward increased expression was observed for samples derived from the transverse colon. Following inflammatory stimulation with LPS, an additional tendency toward increased *COX-2* expression was detected in the transverse colon condition.

Analysis of *IL6* mRNA expression (**Figure 5B**) revealed a significant increase in cells treated with fermentation supernatants derived from the transverse colon, both under basal conditions and after LPS stimulation. A similar upward trend was observed for samples derived from the ascending colon.

#### 3.6.2 *Ex vivo* modulation of inflammatory responses in splenocytes

**Figure 6:**
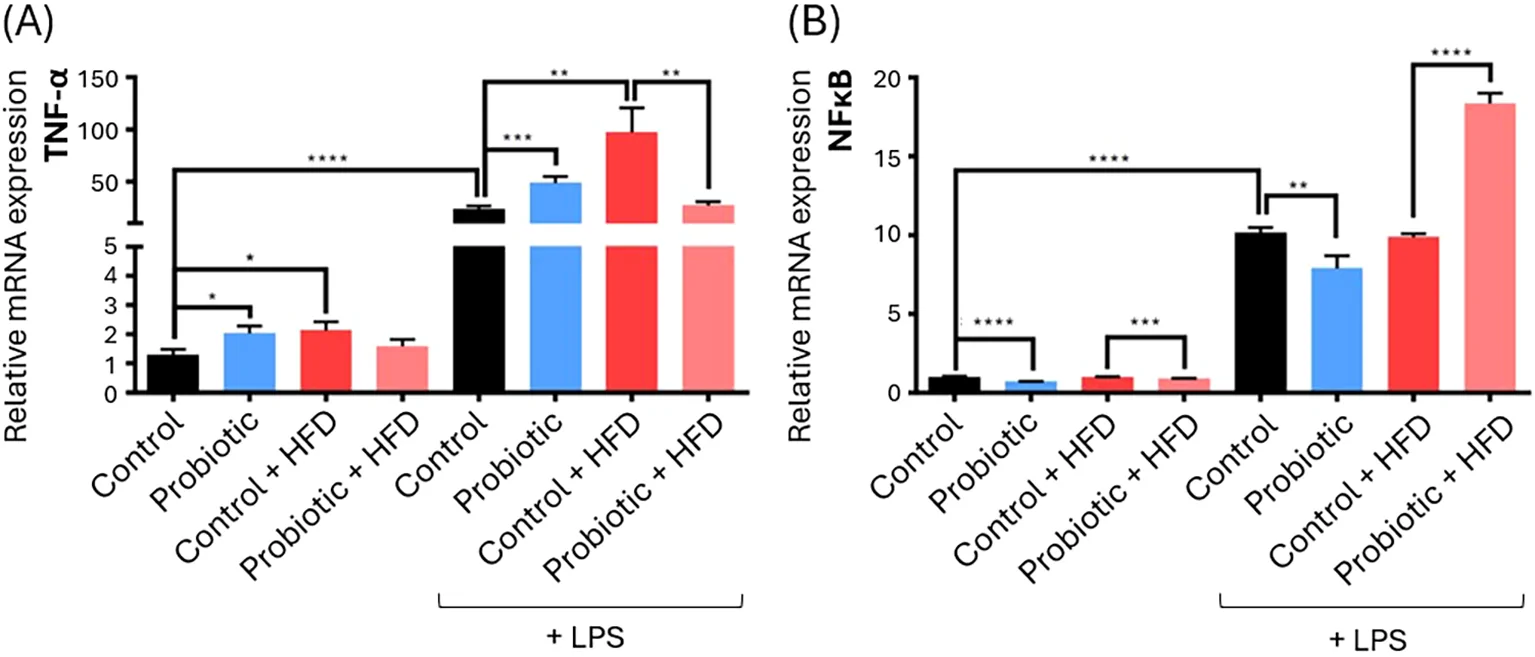
*Ex vivo* expression of inflammatory markers in murine splenocytes following lipopolysaccharide stimulation. Relative mRNA expression of **(A)** tumor necrosis factor (*Tnf*) and **(B)** nuclear factor NF-κB subunit 1 (*Nfkb1*) was determined in splenocytes isolated from mice receiving a standard diet (Control), a standard diet supplemented with *Lactiplantibacillus pentosus* BB (Probiotic), a high-fat diet (Control + HFD), or a high-fat diet supplemented with the probiotic (Probiotic + HFD). Splenocytes were maintained under basal conditions or stimulated with lipopolysaccharide **(LPS)** for 24 h. Gene expression was determined by RT-qPCR, normalized to β-actin (*Actb*), and calculated using the 2^−ΔΔCt^ method. Results are expressed as fold change relative to reference control group (Control). Data are presented as mean ± SD; *n* = 6 biologically independent animals per group. Pairwise comparisons were performed using two-tailed unpaired Student’s *t*-tests. Brackets indicate the groups included in each comparison: *p ≤ 0.05, **p ≤ 0.01, ***p ≤ 0.001, and ****p ≤ 0.0001.

To evaluate the systemic immunomodulatory effects of probiotic supplementation, splenocytes isolated from experimental animals were cultured *ex vivo* and stimulated with LPS to induce an inflammatory response. Relative mRNA expression of *Tnf* (tumor necrosis factor-α) and *Nfkb1* (nuclear factor kappa B) was analyzed as markers of inflammatory activation (**Figure 6**).

As expected, LPS stimulation significantly increased *Tnf* mRNA expression compared with unstimulated cells. Among LPS-treated groups, splenocytes from animals fed the high-fat diet (Control + HFD) exhibited a 319.3 ± 101.7% increase in *Tnf* expression relative to the untreated control group. In contrast, supplementation with *L. pentosus* BB in HFD-fed animals markedly attenuated this response, reducing *Tnf* expression by 303.6 ± 101.9% compared with the Control + HFD group (**Figure 6A**).

Regarding *Nfkb1* mRNA expression, probiotic supplementation significantly reduced basal NFκB levels in animals receiving the standard diet (−30.1 ± 3.2% vs Control). Similarly, in HFD-fed animals, probiotic supplementation resulted in a 10.3 ± 2.9% decrease in *Nfkb1* expression relative to the Control + HFD group under non-stimulated conditions (**Figure 6B**).

Under LPS stimulation, probiotic supplementation alone significantly reduced *Nfkb1* expression by 22.3 ± 7.3% compared with the Control group. In contrast, splenocytes from the Probiotic + HFD group exhibited an 83.8 ± 5.8% increase in *Nfkb1* expression relative to the Control + HFD group following LPS treatment.

## 4 Discussion

The present study combined a dynamic colonic fermentation model with an early high-fat diet (HFD) mouse model to examine whether *Lactiplantibacillus pentosus* CECT 8039 (*L. pentosus* BB) could modulate microbial fermentation and host pathways related to satiety, adipokine regulation, and inflammation. Probiotic treatment was associated with selective changes in representative cultivable microbial groups, substantial increases in acetate, propionate, and butyrate across simulated colonic regions, enhanced enteroendocrine responses *in vitro*, and partial attenuation of HFD-associated weight gain and adipokine disruption *in vivo*. This integrated experimental design links microbial functional outputs with endocrine and systemic outcomes. Nevertheless, the findings demonstrate associations and biological plausibility rather than a complete causal microbiota–metabolite–host pathway.

A major finding was the marked increase in SCFA production following probiotic treatment. Acetate, propionate, and butyrate increased in all simulated colonic compartments, with the largest changes observed in the ascending colon. Propionate showed the most pronounced increase, reaching a 7.34-fold change in the ascending-colon compartment. These findings are consistent with the capacity of probiotics to modify community-level fermentation and microbial metabolite production (11). SCFAs act both as metabolic substrates and signaling molecules and are involved in epithelial, endocrine, immune, glucose, and lipid regulation (10, 14, 15).

In addition to these metabolic changes, *L. pentosus* BB selectively modulated representative cultivable microbial groups within the simulated colonic ecosystem. Lactic acid bacteria increased in the ascending, transverse, and descending colon compartments, whereas *Clostridium* spp. counts decreased across all three regions. Total anaerobic counts remained stable or increased, indicating that these changes were not accompanied by an overall reduction in the cultivable anaerobic population. In contrast, *Bifidobacterium* spp. showed modest reductions of less than 1 log CFU/mL, while Enterobacteriaceae displayed compartment-specific changes. These findings indicate selective and region-dependent modulation rather than a uniformly favorable change across all bacterial groups.

The changes in cultivable microbial populations occurred alongside enhanced SCFA production, but the present study cannot establish whether the observed compositional changes were responsible for the altered metabolite profile. Increased SCFA concentrations may have resulted from direct probiotic metabolism, stimulation or inhibition of resident microorganisms, changes in substrate utilization, or microbial cross-feeding. Consequently, the relationship between the changes in selected microbial groups and SCFA production should be interpreted as descriptive rather than causal.

The increased SCFA concentrations were also associated with enhanced enteroendocrine responses. Fermentation supernatants obtained after probiotic treatment increased GLP-1 release and *Pyy* and *Cck* mRNA expression, although the magnitude and statistical significance of these responses differed among hormones and colonic compartments. Experimental evidence supports a role for SCFAs in anorexigenic hormone regulation. Tolhurst et al. (16) demonstrated that SCFAs stimulate GLP-1 secretion through FFAR2, while Psichas et al. (17) reported propionate-induced GLP-1 and PYY secretion in rodents. Targeted colonic propionate delivery has also been associated with reduced energy intake, attenuated weight gain, and lower adiposity in adults with overweight (18). Furthermore, fermentable carbohydrates may promote FFAR2-dependent expansion and function of colonic PYY-producing cells (19).

These observations provide biological plausibility for an SCFA-associated enteroendocrine response but do not demonstrate that the effects observed in the present study were exclusively mediated by SCFAs or FFAR2/FFAR3 signaling. Whole fermentation supernatants were used in the cellular assays and therefore contained a complex mixture of microbial metabolites. The potential contribution of lactate, bile acid derivatives, indole compounds, or other bioactive microbial products cannot be excluded. Moreover, the relative contribution of individual SCFAs was not examined.

Establishing a causal mechanistic pathway would require treatment of enteroendocrine models with individual SCFAs at concentrations matching those detected in the fermentation samples, together with fractionation or targeted metabolomic characterization of the supernatants. Pharmacological or genetic inhibition of FFAR2 and FFAR3 would also be required to determine whether receptor blockade prevents the GLP-1, PYY, or CCK responses. Equivalent approaches *in vivo* would help establish whether this signaling pathway contributes directly to the observed metabolic phenotype.

The metabolic findings were consistent with a partial attenuation of early HFD-induced dysfunction. Probiotic supplementation reduced HFD-associated weight gain and significantly decreased circulating leptin and resistin levels. The reduction in hyperleptinemia may indicate lower adiposity or attenuation of leptin resistance-associated signaling, although circulating leptin alone cannot distinguish between these possibilities (20). Resistin was also reduced, whereas adiponectin did not change significantly. This differential response argues against a uniform normalization of adipose endocrine function and indicates that the strain may affect distinct adipokine pathways differently.

A more complete assessment of adipose-tissue metabolism would require measurements of adipose-tissue mass, adipocyte size, tissue histology, macrophage infiltration, inflammatory gene expression, and markers of lipogenesis and lipolysis. Insulin concentrations, glucose and insulin tolerance, circulating lipid profiles, energy intake, and energy expenditure would also help determine whether the observed effects were mainly related to appetite regulation, adipose inflammation, substrate metabolism, or a combination of these processes.

Comparison with the broader probiotic literature should take into account the strong strain specificity of probiotic effects. Meta-analyses suggest modest average effects of probiotic supplementation on body weight and adiposity, but outcomes vary according to strain, dose, treatment duration, host characteristics, and background diet (21, 22). Previous comparative studies have similarly indicated that weight-related effects cannot be generalized across probiotic species or strains (23).

A distinctive feature of the present study is the integration of regional colonic fermentation, microbial-group enumeration, enteroendocrine cellular responses, and metabolic and immune outcomes in an HFD model. This combined approach provides a strain-specific functional profile characterized by increased microbial fermentation, enhanced enteroendocrine responses, and selective modulation of leptin, resistin, and inflammatory markers. However, the study did not directly compare *L. pentosus* BB with other probiotic strains. Therefore, superiority or a unique advantage over established metabolic probiotics cannot be concluded without head-to-head comparative studies.

The immune findings were heterogeneous and should not be interpreted as uniformly anti-inflammatory. Increased *IL6* and *PTGS2* expressions were observed under selected Caco-2/THP-1 conditions following exposure to fermentation supernatants. These responses may reflect epithelial or innate immune activation, but the present assays cannot determine whether they represent beneficial immune priming, transient cellular stress, or a potentially adverse pro-inflammatory response.

Similarly, reduced basal *Nfkb1* mRNA expression and attenuated *Tnf* mRNA expression in splenocytes contrasted with increased *Nfkb1* expression after LPS stimulation in the Probiotic + HFD group. Since NF-κB regulates immune activation, cellular survival, and stress responses in addition to inflammation, this pattern is more appropriately interpreted as stimulus- and context-dependent immunomodulation than as generalized inflammatory suppression. Furthermore, because the cellular assays were performed using complete fermentation supernatants, the observed immune effects cannot be attributed specifically to SCFAs or to any individual microbial metabolite.

The molecular connection between intestinal microbial activity and systemic immune modulation remains unresolved. SCFAs can regulate epithelial tight-junction assembly and immune-cell function. Butyrate, for example, has been reported to enhance epithelial barrier integrity through AMPK-dependent tight-junction assembly (24), while broader SCFA-mediated effects on epithelial and immune homeostasis have been extensively described (25, 26). However, intestinal permeability, tight-junction proteins, mucosal cytokines, endotoxemia markers, and intestinal immune-cell populations were not assessed in the present study. These measurements, integrated with comprehensive microbiota and metabolite profiling, would be required to connect local intestinal regulation with systemic splenocyte responses.

The dynamic fermentation platform reproduces relevant regional conditions, including pH, retention time, anaerobiosis, and microbial metabolism, but it cannot reproduce the complete *in vivo* interactions among microbiota, intestinal epithelial cells, enteroendocrine cells, immune cells, neural pathways, and the vascular system. The *in vitro* and *in vivo* experiments therefore provide complementary evidence but do not constitute direct validation of one another, as they were performed as separate models and did not evaluate identical mechanistic endpoints.

Human intestinal organoids, organoid-derived monolayers, and advanced epithelial–immune co-culture systems could model selected host–microbiota interactions more directly (27). Future studies combining donor-specific fermentation samples with human intestinal organoids, immune-cell co-cultures, and expanded animal endpoints would improve translational confidence while preserving control over microbial and metabolite exposures.

A further limitation is the absence of comprehensive microbiota profiling. Microbial composition was assessed by selective culture-based enumeration of five representative groups: *Bifidobacterium* spp., lactic acid bacteria, Enterobacteriaceae, *Clostridium* spp., and total anaerobes. Although this approach provides information on selected viable and cultivable populations, it cannot characterize alpha diversity, beta diversity, global community structure, or changes in uncultivated taxa. It is therefore not possible to comprehensively identify which microorganisms contributed to the increased SCFA production, whether *L. pentosus* BB persisted within the fermentation system, or whether microbial cross-feeding networks were modified.

Future studies incorporating 16S rRNA gene sequencing or shotgun metagenomics, together with strain-specific tracking and metabolomics, would substantially strengthen the interpretation of the microbiota findings. The limited number and characterization of fecal donors also restrict generalizability, as donor-dependent variability can substantially affect microbial composition and fermentation responses.

Additional limitations include the short intervention period, small animal-group size, use of male mice only, incomplete characterization of food and energy intake, reliance on relative fold changes for several outcomes, and limited methodological detail for some analytical procedures. These factors constrain reproducibility, statistical robustness, and causal interpretation. Conclusions should therefore remain focused on the observed associations and avoid extrapolation to long-term efficacy or clinical benefit.

Future studies should combine targeted interventions with individual SCFAs and FFAR2/FFAR3 blockade with comprehensive metabolomic and microbiota profiling. Assessment of intestinal permeability, tight-junction proteins, mucosal cytokines, immune-cell populations, adipose-tissue remodeling, lipid metabolism, glucose homeostasis, food intake, and energy expenditure would help connect local microbial activity with systemic metabolic and immune outcomes (28). Validation in human intestinal organoid-based models, adequately powered and sex-balanced animal studies, donor-stratified fermentation experiments, and ultimately controlled human interventions will be required to establish causality and translational relevance.

Overall, the data indicate that *L. pentosus* BB modifies selected cultivable microbial populations and microbial fermentation activity and that these changes are associated with increased SCFA concentrations, enhanced enteroendocrine signaling, and partial attenuation of early HFD-associated metabolic dysfunction. The evidence is strongest for a functional association across complementary experimental models. Confirmation of an SCFA–FFAR2/FFAR3 causal axis, identification of the microorganisms and metabolites responsible for the observed responses, and demonstration of intestinal-barrier and adipose-tissue effects remain priorities for future research.

## Data Availability

The datasets presented in this article are not readily available because the raw data are not publicly deposited. Requests to access the datasets should be directed to the corresponding authors (vnavarro@ucam.edu or ialberola@laespanola.es) and will be considered upon reasonable request with a duly justified reason.
